# COVID-19 and the Kidneys: An Update

**DOI:** 10.3389/fmed.2020.00423

**Published:** 2020-07-21

**Authors:** Claudia Benedetti, Meryl Waldman, Gianluigi Zaza, Leonardo V. Riella, Paolo Cravedi

**Affiliations:** ^1^Department of Medicine, Icahn School of Medicine at Mount Sinai, New York, NY, United States; ^2^Kidney Disease Branch, National Institute of Diabetes and Digestive and Kidney Diseases, National Institutes of Health, Bethesda, MD, United States; ^3^Renal Unit, Department of Medicine, University Hospital of Verona, Verona, Italy; ^4^Renal Division, Harvard Medical School, Massachusetts General Hospital, Boston, MA, United States

**Keywords:** acute kidney injury, AKI, COVID-19, dialysis, transplant

## Abstract

The new coronavirus disease 2019 (COVID-19) has become a world health emergency. The disease predominantly effects individuals between 30 and 79 years of age with 81% of cases being classified as mild. Despite the majority of the general population displaying symptoms similar to the common cold, COVID-19 has also induced alveolar damage resulting in progressive respiratory failure with fatalities noted in 6.4% of cases. Direct viral injury, uncontrolled inflammation, activation of coagulation, and complement cascades are thought to participate in disease pathogenesis. Patients with COVID-19 have displayed kidney damage through acute kidney injury, mild proteinuria, hematuria, or slight elevation in creatinine possibly as consequence of kidney tropism of the virus and multiorgan failure. The impact of COVID-19 on patients with pre-existing kidney impairment, including those with chronic kidney disease, kidney transplant recipients, and individuals on hemodialysis (HD) has not yet been clearly established. No specific treatments for COVID-19 have been found yet. Research has revealed several agents that may have potential efficacy against COVID-19, and many of these molecules have demonstrated preliminary efficacy against COVID-19 and are currently being tested in clinical trials.

## Introduction

Severe acute respiratory syndrome coronavirus 2 (SARS-CoV-2), first described in humans in December 2019 in Wuhan, China (1), is the third coronavirus to have emerged in the last 20 years. Previous outbreaks of the severe acute respiratory syndrome coronavirus (SARS-CoV) in 2002 and the Middle East respiratory syndrome coronavirus (MERS-CoV) in 2012 have been toppled in case incidence by the global impact of SARS-CoV-2 (2).

As of May 25, 2020, 5,370,375 infected cases have been confirmed with 344,454 deaths across 216 countries, areas or territories (3). SARS-CoV-2 was declared a pandemic on March 11, 2020 by the World Health (1).

Presentation of symptoms for COVID-19 infection can be seen 2–14 days after exposure. These symptoms include fever, cough, and difficult breathing (4, 5). A severe complication of this disease is progressive respiratory failure, and death may occur in 6.4% of the cases (3, 6, 7). The potential impact of SARS-CoV-2 on the kidneys is still undetermined, but emerging evidence indicates that kidney complications are frequent, and COVID-19 disease may have unique features in individuals on chronic dialysis and kidney transplant recipients (8).

## SARS-CoV-2 and COVID-19 Pathophysiology

The coronavirus derives its names from its physical form: a spherical virion with spike (S) proteins (9). There are four subfamilies of coronaviruses, namely α-, β-, χ-, and δ-coronaviruses; SARS-CoV-2 is a β-coronavirus like its predecessor, SARS-CoV (9). The relationship between the current strain and previously identified strains through high-throughput sequencing has allowed epidemiologists to trace the evolution of the virus. A closely related strain found in bats, RaTG13-2013, shares 96% of the genome (4). This has led scientists to believe that the virus jumped between host species from the bat, or an intermediate animal, before spreading amongst the human population (4).

**Figure 1:**
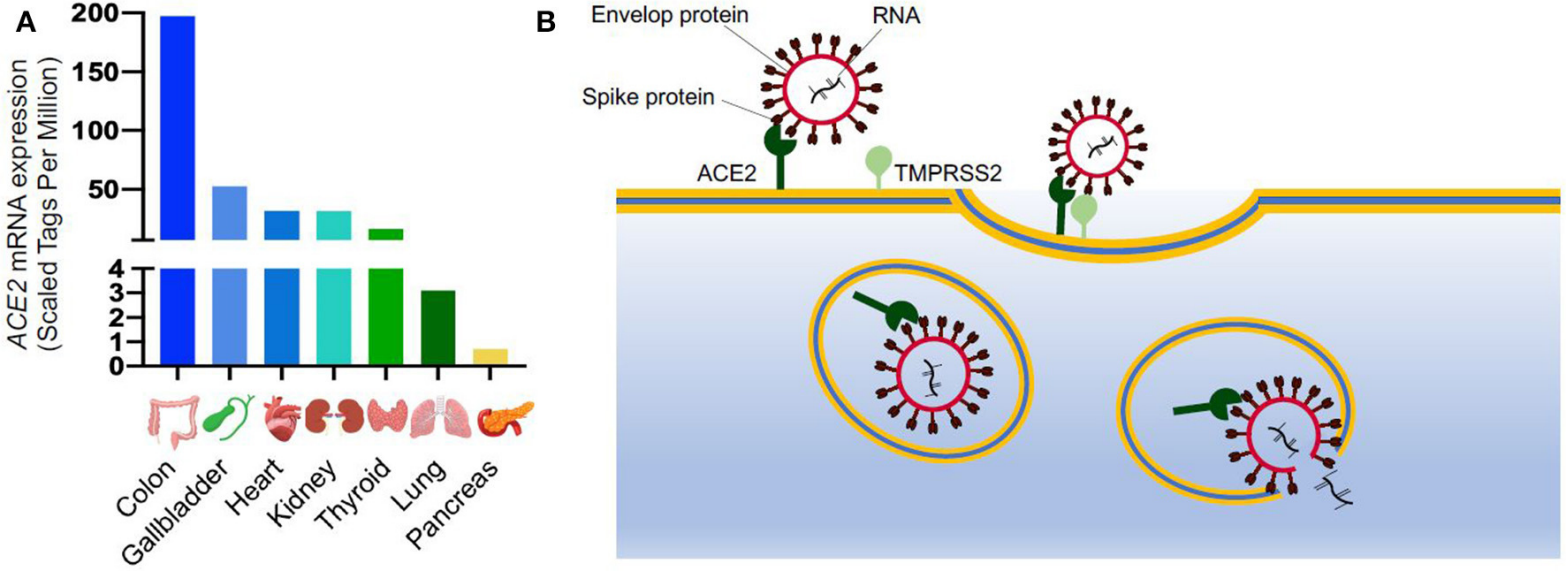
SARS-CoV-2 entry into the cells. **(A)** ACE2 mRNA expression in different organs from FANTOM5 dataset (11); **(B)** Schematic of SARS-CoV-2 entry into the cells. ACE2 is expressed on cell surface and it is recognized by the spike protein of SARS-CoV-2. After binding to ACE2, the viral spike glycoprotein is primed by a host serine protease (TMPRSS2), which allows internalization by endocytosis. Once inside the cells, SARS-CoV-2 replicates utilizing the cellular transcriptional machinery. ACE2, Angiotensin-Converting Enzyme 2; SARS-CoV-2, Severe Acute Respiratory Syndrome Coronavirus 2; TMPRSS2, Transmembrane Serine Protease 2.

The spike protein is key to its high-virality as the RNA virus enters cells through binding between the S protein and its host receptor. The virus efficiently binds to the angiotensin converting enzyme 2 (ACE2) receptor (10) which is highly expressed in many organs including the bronchus and lung parenchyma, heart, kidney, and gastrointestinal tract (Figure 1A) (6, 12, 13). Zhao et al. demonstrated that ACE2 is highly expressed in the alveolar epithelial type II cells (AECII), suggesting that these cells could be the reservoir for the virus. Other studies have showed that the AECII cells have several genes related to the viral process, replication, life cycle and assembly, therefore facilitating the viral replication in the lung (10).

After binding to ACE2, the cellular transmembrane protease, serine 2 (TMPRSS2) mediates the S protein priming allowing the virus to enter the host cells through clathrin-dependent endocytosis (Figure 1B) (13). The endosomal entry way for the virus requires a low inner pH, and once inside, the virus exploits the cellular transcriptional machinery to replicate itself and spread throughout the host (8). By hijacking the endogenous transcriptional machinery, the virus alters the behavior of the host cells and tissue making them unable to fulfill their normal function.

There are two phases to the immune response induced by SARS-CoV-2 (14, 15): (1) an initial specific adaptive immune response and (2) uncontrolled inflammation. The adaptive response is required during the early stages of incubation to prevent the progression of disease and eliminate the virus. When the protective immune response is ineffective, the virus propagates, inducing destruction of the affected tissues leading to severe disease progression (15). An uncontrolled inflammatory response is also implicated in COVID-19, as a mechanism responsible for acute respiratory distress syndrome (ARDS). The release of a cytokine storm may promote apoptosis or necrosis of T cells, and consequently lead to their reduction (16). This violent assault can be seen in patients with severe disease who's plasma levels of IL-6, TNFα, and IL-10 are higher, and circulating CD4 and CD8 T cells are lower than patients with mild COVID-19 or healthy controls (17). This trend is even stronger in elderly patients. Intriguingly, most severe COVID-19 cases also have increased percentages of T cells with exhaustion phenotype. Therefore, it is tempting to speculate that uncontrolled inflammation, while promoting ARDS, impairs viral clearance by inducing T cell exhaustion (18).

### Complement Activation in COVID-19

The complement system is an important component of innate immunity that is essential to respond rapidly to infection. During inflammation, both acute and chronic, activation of the complement system promotes the elimination of pathogens. Dysregulation of the complement system may lead to acute lung disease after a highly pathogenic viruses infections (19).

Complement activation through the lectin pathway has already been described in SARS-CoV-2 infected patients. Gao et al. (20) showed that blocking this pathway ameliorates lung injury induced by SARS-CoV and MERS-CoV in mice. Autoptic lung samples and skin biopsies from patients with severe COVID-19 showed a deposition of mannose binding lectin (MBL)-associated serine protease (MASP)2, C4d, and C5b-9 (MAC component), suggesting an activation of the complement system through the alternative and the lectin pathways (21). Preliminary data from patients treated with an anti-complement C5a blocking antibody also suggested a potential benefit of complement targeting therapies in COVID-19 patients with severe lung injuries (20). Due to the implicated role of complement in the pathogenesis of acute lung injury and ARDS, ongoing clinical trials are testing the hypothesis that blocking the complement cascade ameliorates disease severity in COVID-19 patients.

### Hypercoagulability and Thrombosis in COVID-19

In general, altered hemostasis due to viral infections often leads to vascular complications like thrombosis and or hemorrhage. Vascular and endothelial changes by the virus as well as inherited host factors help define the clinical presentation. Some viral contributions to the ischemic and thrombotic environment include procoagulant factors, hemodynamic changes, and pro-inflammatory cytokines. The pro-inflammatory response also directly induces plaque rupture (22–25). This can be seen with many respiratory viruses like H1N1 influenza (26) and is of increasing concern for patients with severe COVID-19. The majority of severe COVID-19 patients show signs of increased coagulation activity, resulting in consumption of coagulation factors and disseminated microvascular thrombosis. Hypoxia associated with COVID-19 pneumonia increases blood viscosity both directly and through hypoxia-inducible transcription factor-dependent signaling pathway thereby promoting thrombosis (27). Laboratory results indicate a prolongation of the prothrombin time and international normalized ratio (PT/INR) and partial thromboplastin time (PTT), elevation of D-dimer, decreased fibrinogen levels, thrombocytopenia, and schistocytes in peripheral blood smears. In one study of 449 COVID-19 patients, 71% of non-survivors had diffuse intravascular coagulation (DIC) compared to 0.4% of survivors (27). Zhou et al. (25) showed that death is associated with D-dimer <1 μg/mL in a retrospective study of 191 COVID-19 patients. Elevated D-dimer at admission and markedly increasing D-dimer levels during the disease were associated with high mortality (27).

Recently, anti-phospholipid antibodies and infarcts in multiple vascular territories have been reported in three COVID-19 patients with thrombocytopenia. It is known that these antibodies can increase during several infections, and critical illness and may lead to thrombotic events (28).

Based on this data, a prophylactic dose of low molecular weight heparin has been advised to hospitalized COVID-19 patients, despite abnormal coagulation tests, and the thrombotic risk associated with prolonged bed resting (27).

## COVID-19 Disease Natural History

SARS-CoV-2 rapidly spread throughout the world since its first case and very few countries to date have not reported at least one case of the disease within their borders. One infected patient may transmit the virus to 2 or 3 other individuals, and asymptomatic patients may also spread the virus (29, 30). The proportion of this asymptomatic population remains unknown (4). Transmission of the SARS-CoV-2 occurs through direct human-to-human contact and through respiratory droplets in the air or on surfaces (31). The virus remains viable in aerosols for over 3 h without a significant reduction in its infectious titer and for up to 72 h on plastic and stainless steel (32, 33).

Approximately 80% of patients infected by SARS-CoV-2 present with mild disease and can recover spontaneously (34). The other 20% of patients present with severe disease and 6% become critically ill (35). In these symptomatic patients, the main signs manifest as an upper respiratory tract infection, cough, fever, and asthenia. Patients with severe disease will present with pneumonia suspected by the presence of decreased oxygen saturation, lymphopenia and increased inflammatory markers (CRP, D-dimer, ferritin) (4, 36). Chest CT scans usually show bilateral involvement of the lungs, with consolidation in subsegmental areas (16). Viral pneumonia can evolve into severe acute hypoxic respiratory failure mediated by increased pulmonary capillary permeability and damage of the alveolar epithelial cell (35).

The main risk factor for mortality due to COVID-19 is advanced age. In patients with comorbidities, such as chronic kidney disease, hypertension, chronic obstructive pulmonary disease, diabetes, tumor, and obesity, advanced age was the strongest predictor of a poor outcome with infection (33, 35, 37). Male sex has also been proposed as a risk factor in some studies (1), but this is not an universal finding (38).

## Kidney Involvement During COVID-19

Li et al. (39) analyzed kidney function in 193 COVID-19 patients and found that 31% of patients had an elevated level of blood urea nitrogen (BUN) and 22% had increased serum creatinine. These authors also found also that in 147 patients, 60% exhibited proteinuria and 48% exhibited hematuria (not peer-reviewed) (39). D-dimer was elevated in 70% of 182 patients and high levels of D-dimer were common in severe and deceased cases (39). In another retrospective study of 333 patients, about 75% experienced urine dipstick abnormalities or AKI (40). In the first studies during the pandemic spread, incidence of AKI was reported from 3 to 9% of patients, but more recent studies reported an incidence rate of 15% (17, 41). In one observational study of 5,449 hospitalized patients, the incidence of AKI was 36.6 with 14.3% of patients requiring dialysis and was even higher in patients admitted to the ICU (42). Moreover, patients with AKI had higher mortality compared to those without AKI (35 and 16.3%, respectively) (42).

AKI is more common among patients with more severe disease, particularly in those recovering in the ICU, and is considered a negative prognostic factor for survival (43). In a retrospective study of 333 patients with COVID-19 pneumonia, those who presented with kidney dysfunction had higher mortality rates than patients without kidney involvement (11.2 and 1.2%, respectively) (40).

In a single-center, retrospective, observational study of 287 patients, 55 patients presented with AKI as defined by the Kidney Disease Improving Global Outcomes (KDIGO). These patients were significantly older, more likely male, and with other comorbidities, including chronic renal insufficiency, hypertension, and cerebrovascular disease, and tended to have more severe pneumonia (not peer-reviewed) (41). Of these patients, 14.3% presented with AKI at stage 1, while 4.9% of patients presented with stage 2 or 3 (41). A comparison of outcomes according to AKI status and stage found that comparing non-AKI patients to those who developed AKI, the last had higher mortality rates, especially when AKI was severe (mortality: 3.0, 7.3, and 64.3% for non-AKI, AKI stage 1, and AKI stages 2/3, respectively) (41).

In another study of 701 patients, 11.9% of those with elevated baseline creatinine developed AKI compared to 4.0% in patients with normal baseline creatinine (5). In-hospital mortality was significantly higher in patients with proteinuria, hematuria, elevated baseline creatinine and urea, and AKI stage 2–3 (5).

High dose diuretics and K-binding resins have been used to delay the need of dialysis (44–46). Careful fluid management to reduce the risk of pulmonary edema in patients with severe ARDS from COVID-19 is the first goal, so in the absence of hypotension and shock, a negative fluid balance of 0.5–1.0 L per day is recommended (47).

The current management of COVID-19 associated AKI includes supportive treatment, avoiding nephrotoxic drugs, and early start, when possible, of renal replacement therapy (5). SARS, MERS, and sepsis have been successfully treated in the past with continuous renal replacement therapy (CRRT). In these cases, CRRT by hemofiltration and hemodiafiltration can contribute to the improvement of organ failure. Therefore, CRRT may be beneficial in patients with COVID-19 and sepsis syndrome, but it needs to be evaluated more carefully (48). Filters with membranes made of acrylonitrile and sodium methallyl sulfonate plus polyethyleneimine or polymethylmethacrylate could adsorb cytokines, but they should be replaced every 24 h (49). Finally, the new sorbent cartridges designed to remove circulating cytokines and mediators, associated with hemoadsorption and hemoperfusion, could also be considered in COVID-19 patients (50).

Prone positioning, already known to reduce mortality in other causes of severe ARDS, should be applied early. Although there is no available data on patients with COVID-19 (33), SARS-CoV-2 tends to affect the peripheral and dorsal areas of the lungs. Prone positioning could potentially improve the response to a positive oxygenation (33). This, however, complicates placement of a central venous catheter and could hinder the ability to do CRRT for increased hemodynamic instability.

Due to the hypercoagulability state associated with COVID-19, systemic anticoagulation with unfractionated heparin or regional citrate anticoagulation is advised (46). Since these patients experience higher filter clotting, it might be useful to choose predilution replacement fluid administration for hemofiltration, and consider using a heparin bolus together with pre-filter heparin at higher rate than usual -monitoring PTT before and during hemodialysis to avoid bleeding- in order to maintain higher blood flows (46). It is also important to consider that most COVID-19-infected patients requiring intensive care management show altered liver function which associates with an increased risk for citrate accumulation (51).

In a single-center retrospective observational study of 287 patients, Xiao et al. (41) found that most patients recover from AKI stage 1. However, patients who progress to AKI stage 2 or 3 have a very high mortality rate (41). The impact of COVID-19 on patients' long-term kidney function should be investigated further.

### The Mechanisms of SARS-CoV-2 Associated Kidney Injury

The exact mechanism of kidney involvement is unclear and likely multifactorial. Kidney disease may be caused by SARS-CoV-2 binding to the ACE2 receptor on kidney cells that allows the virus to enter (13, 52, 53). Moreover, normal kidney and intestinal tract have higher ACE2 expression than lung tissue (54). Detection of coronavirus in the kidneys and urine of patients with SARS-CoV and SARS-CoV-2 supports the theory that the virus can directly damage the kidneys (5). However, in another study, no urine tested positive for viral RNA in 72 samples (55). Preliminary evidence in postmortem examinations of kidney tissue from six patients found severe acute tubular necrosis and lymphocyte infiltration. Additionally, SARS-CoV-2 nucleocapsid protein (NP) has been detected through immunohistochemistry in kidney tubules (56). Moreover, in one autopsy of a kidney transplant patient who died of COVID-19, viral inclusion structures were found in the endothelial cells of the kidney (57). Viral infection could induce tubular damage through the deposition of the MAC complex (the final step of the complement cascade) on tubules and infiltration of CD68^+^ macrophages in the tubule-interstitium (56). Diffuse damage in proximal tubules with the loss of brush border, vacuolar degeneration, and even necrosis were observed in a study of 26 autopsies of patients with COVID-19 (58). In the peritubular and glomerular capillary of these autopsies, diffuse erythrocyte aggregation with endothelial damage, and obstruction without fibrin thrombi or distinct fragmentation of erythrocytes or platelets were observed (58). Clusters of SARS-CoV-2 was found with electron microscopy in the tubular epithelium and podocytes (58). However, this finding could be non-specific as the presence of viral proteins may not represent direct damage mediated by the virus and instead indicate clathrin-coated vesicles (59, 60). Puelles et al. (61) showed the presence of SARS-CoV-2 RNA and proteins in all kidney areas, especially in glomerular cells in autopsies of three of six COVID-19 patients.

Kidney biopsies in two Afro-American patients with high-risk APOL1 genotype and COVID-19 infection showed a collapsing focal segmental glomerulosclerosis (62, 63). However, SARS-CoV-2 RNA was not detected in the kidney tissue raining the intriguing hypothesis that cytokine storm increased APOL1 expression leading to podocyte injury (62, 63). Other indirect mechanisms that potentially lead to tubular injury are sepsis, cytokine storm syndrome, shock/hemodynamic instability, rhabdomyolysis, and hypoxia of kidney tissue (5, 48).

Kidney biopsies would be helpful to better understand the histologic pattern of injury (tubular, glomerular, and vascular) and the pathogenesis that could led to AKI. Unfortunately, this is very difficult to obtain given the respiratory and hemodynamic instability of AKI patients and the use of anticoagulation which increases the risk of bleeding. In addition, non-essential procedures in infected patients are not being done in most hospitals given significant risk of exposure to personnel.

### COVID-19 in CKD Patients

Patients with chronic kidney disease (CKD) are known to have a higher risk of upper respiratory tract infection and pneumonia due to their persistent proinflammatory state with functional defects in innate and adaptive immunity (5). No study so far has found that chronic kidney disease is statistically correlated with severe COVID-19. However, a significant association of CKD with severe COVID-19 was observed when data of different studies were combined (64).

### COVID-19 in Kidney Transplant Recipients

Chronic immunosuppression is a well-known risk factor for viral and bacterial infections, but it is also crucial to prevent graft rejection and to contrast the uncontrolled antiviral inflammatory response. Therefore, the transplant community is puzzled in trying to understand the best therapeutic approach, in the absence of any strong clinical data (8).

So far, initial presentation in transplant recipients has been reported heterogeneous to other hosts and many patients did not report contact with infected individuals. Common symptoms at disease onset have been fever, cough, asthenia, myalgias, and diarrhea (65). In a study of 36 transplant recipients, however, fever was less common than in general COVID-19 patients (66). In multiple series, transplant patients show numerous radiopacity and patchy shadows on chest radiographs often at presentation (67–71). However, in one case series of 15 kidney transplant patients, 33% had no acute radiographic findings (72). Laboratory exams often showed lymphopenia with lower CD3, CD4, and CD8 T cells especially in those patients who had received antithymocyte globulin in the weeks before the infection (66).

Until more data is available, the rules to prevent viral infection in the general population apply to transplant patients (hand hygiene, sanitization, social distancing, and avoiding areas where infected patients could be present) (73). Transplant patients with potential COVID-19 infection should not access the transplant center due to risk of viral spread.

According to the European Renal Association—European Dialysis and Transplant Association (ERA-EDTA) guidelines (74), in patients with COVID-19 and without pneumonia, complete withdrawal of immunosuppressants -particularly calcineurin inhibitors (CNI)- is discouraged. Reduction of the dosage of CNI, and withdrawal of mycophenolate, azathioprine, or mTOR-inhibitors should be individualized considering the severity of the disease (74). The concurrent use of antivirals and anti-inflammatories should be carefully considered with attention to drug-drug interactions that may affect the half-life of immunosuppressant drugs (74).

In critically ill patients, withdrawal of immunosuppression could be done while converting those patients to hydrocortisone/solumedrol. This approach may improve viral clearance but could lead to immune reconstitution and kidney's rejection (69, 75). It should be considered that reducing immunosuppression may exacerbate inflammation, so this approach should be cautioned in the absence of anti-inflammatory agents (see below).

In some patients, tacrolimus reduction may be preferred over complete withdrawal (67, 69) because of direct alleged CNI antiviral properties or CNI anti-inflammatory action (76, 77). Pending the results of clinical studies, CNI withdrawal vs. reduction may be established on a case-by-case basis depending on the severity of pneumonia.

#### Living Kidney Donation During the COVID-19 Pandemic

In countries with widespread community transmission, living-donor kidney programs have been temporarily suspended. In countries where community transmission is lower, living donations should not be performed if the donor or recipient have lived in a place with high incidence or have been in contact with confirmed or suspected COVID-19 patient within 14 days.

Transplantation can be considered in highly selected cases when required as a life-saving procedure.

#### Donation From Deceased Donors During the COVID-19 Pandemic

In countries with sporadic COVID-19 infection, deceased donor transplants should continue. However, donors at risk of infection should not be accepted since RNAemia was reported in at least 15% in one case series, and, transmission from the donor is possible (16).

Suspension of all transplants that require T or B cell depletion (i.e., hyperimmune patients with greater title of donor-specific HLA antibodies) should be considered even in countries where the incidence of COVID-19 positive individuals is low.

In countries with widespread infection, temporary suspension of the deceased donor program for non-life-saving organs should be considered in order to prevent infection of the recipient during the post-transplant period. Even then, each kidney transplant should be considered case-by-case.

### COVID-19 Infection in Chronic Dialysis Patients

Although it is known that diabetic nephropathy is an important comorbidity and AKI is one of the main risk factors for poor outcome during COVID-19 infection, the impact of the infection on other kidney diseases, like end-stage renal disease, is still unclear (8).

Wang et al. (78) described the outbreak of COVID-19 in the hemodialysis (HD) center of Renmin Hospital, Wuhan University. They identified COVID-19 in 37 individuals among 230 HD patients (16.09%) and 4 individuals among 33 staff (12.12%). They presented mostly mild symptoms, and no one required admission to the ICU. During the observation, 7 HD patients died −6 with COVID-19 and 1 without- but deaths were not directly related to pneumonia. The causes of death, in fact, were heart failure, hyperkalemia, and cerebrovascular disease (not peer-reviewed) (79). In a report of five HD patients, diarrhea was the most common symptom, whereas fever, cough, and dyspnea were not present, thus making the diagnose harder (80). In one retrospective multicenter study of 7,154 hemodialyzed patients, about 2% were confirmed having COVID-19 infection and only about 50% of them presented fever while about 20% of patients were asymptomatic (81). The mortality rate among these patients is greater than the general population with COVID-19 peaking at about 31% (81). The same mortality rate was reported in another retrospective study of 59 dialyzed patients (2 on peritoneal dialysis and 57 on hemodialysis) (82).

Circulating CD4 and CD8 T cells, NK cells, and proinflammatory cytokines are significantly lower in COVID-19 HD patients, compared to non-HD COVID-19 individuals (79). Consistently, HD patients infected by SARS-CoV-2 are more likely to present mild symptoms with lower risk of developing ARDS compared to COVID-19 patients not on HD (79). However, the reduced inflammatory response in HD patients suggests that they may be at higher risk of being infected with SARS-CoV-2. Therefore, additional prevention measures are essential in managing the epidemic in HD centers (8). The impaired immune response in HD patients correlates to longer time to clear the virus requiring longer time in isolation corresponding with the outbreak of SARS in 2003 (79, 83).

An interim guidance for outpatient HD facilities has been recently released by the Centers for Disease Control and Prevention (CDC) (84). Early recognition and isolation of individuals with respiratory infection, isolation of infected patients from other hemodialyzed patients, and the use of personal protective equipment are high priority (85). During routine clinical visits, face masks, and eye shields are sufficient, while during high-risk procedures, N95 respirators and other respiratory protection devices are required. Chinese Society of Nephrology and the Taiwan Society of Nephrology have recently published detailed guidelines for managing COVID-19 outbreaks in dialysis units (8, 48).

Patients on peritoneal dialysis should be managed from home, using telemedicine assistance or other systems for communication whenever possible (86).

## COVID-19 Treatment

Currently, no specific treatment against SARS-CoV-2 has been developed, but research thus far has revealed several agents that may have potential efficacy against COVID-19 (Table 1). Several broad-spectrum antiviral drugs, already approved for other viral infections, are now being tested to treat COVID-19. Meanwhile, anti-inflammatory drugs are given to prevent ARDS. The use of antiviral therapy should be applied early during the disease, when anti-inflammatory therapy, like corticosteroids, could be harmful and induce viral replication. However, once the disease is advanced and the hyper inflammation is the driver of the disease, the use of anti-inflammatories is suggested, while antiviral therapy could be ineffective (107).

**Table 1:** Main treatments, currently in use, and under investigation, in COVID-19 patients.

**Main drugs in use**	**Mechanism of action**	**Comment**
**Antivirals**		
Lopinavir/Ritonavir	Protease inhibitor	A RCT showed no benefit compared to control group (87)
Chloroquine/Hydroxychloroquine ± azithromycin	Increases endosomal pH	Not reduced risk of death in a study of 1,376 patients (88)
Favipiravir	RNA-dependent RNA polymerase inhibitor	Improved symptoms but not higher recovery rate in a RCT (89)
Remdesivir	Nucleoside analog inhibitors	One RCT showed faster recovery, another did not evidence clinical improvement (90, 91)
Convalescent plasma	Plasma with anti-SARS-CoV2 Ab	Effective and safe in some case reports (92)
**Anti-inflammatories**		
Steroids	Broad anti-inflammatory effects, including inhibition of multiple cytokine expression	Increased death in a retrospective study on 548 patients (93). In a retrospective study on 201 patients methylprednisolone was beneficial in those with severe disease (94)
IVIg	Block FcR activation	Only retrospective studies or case reports (95)
Tocilizumab	Recombinant humanized anti-IL-6R monoclonal Ab	Improved symptoms in retrospective studies (96)
Anakinra	IL-1 receptor antagonist	Clinical improvement in a retrospective study (97)
Eculizumab	Humanized anti-C5 monoclonal Ab	Ongoing trials
**Others**		
Stem Cells Therapy	Anti-inflammatory and immune regulatory effects	Only case reports. Numerous ongoing trials
Heparin	Anticoagulant	Improved hypoxia in a case series (not peer-reviewed) (98)
**Drugs under investigation**		
**Antivirals**		
Camostat mesilate	TMPRSS2 inhibitor	Ongoing trials
Arbidol (Umifenovir)	Inhibitor of virus-mediated fusion with target membrane	Not improvement in a retrospective study (99)
Bromhexine hydrochloride	Transmembrane protease serine inhibitor	Ongoing trials
Danoprevir	HCV NS3 protease inhibitor	Efficacy proved in a small open clinical trial (not peer-reviewed) (100)
Interferons	Broad spectrum antivirals	RCT proved benefit in triple therapy (101)
Nitric Oxide Gas	Inhibits viral protein and RNA synthesis	Ongoing trials
Oseltamivir	Viral neuraminidase inhibitor	No effective outcomes in a retrospective study on 138 patients (102)
**Anti-inflammatories**		
Baricitinib	JAK/STAT inhibitor	Clinical improvement in a case series (103)
Bevacizumab	Monoclonal anti-VEGF Ab	Ongoing trials
Clazakizumab	Humanized monoclonal anti-IL-6 Ab	Ongoing trials
Colchicine	Inhibition of the assembly of the NLRP3 inflammasome	Ongoing trials
Fingolimod	Sphingosine-1-phosphate receptor regulator	Ongoing trials
Naproxen	Inhibitor of both COX-2 of Influenza A virus NP	Ongoing trials
Pirfenidone	Inhibits IL-1β and IL-4	Ongoing trials
Ruxolitinib	JAK 1 and JAK 2 inhibitor	Ongoing trials
Sarilumab	Recombinant human anti-IL6R monoclonal Ab	Preliminary data from an ongoing RCT showed decreased inflammatory markers in treatment group
Siltuximab	Anti-IL-6 chimeric monoclonal Ab	Decrease inflammatory markers in a retrospective study (104)
Thalidomide	Reduces TNFα	Ongoing trials
Ulinastatin	Reduces TNFα, IL-6, and IFN-γ and increases IL-10	Ongoing trials
**Others**		
Losartan	Angiotensin II receptor blocker	Safe according to large retrospective studies (105, 106)
Carrimycin	Macrolide antibiotic	Ongoing trials
Vitamin C	Antioxidant properties	Ongoing trials

*Ab: antibody; COX-2: cyclooxygenase-2; HCV: hepatitis C virus; IL-6R: interleukin-6 (IL-6) receptor; NLRP3: NLR Family Pyrin Domain Containing 3; RCT: randomized controlled trial; TMPRSS2: transmembrane serine protease 2; TNFα: tumor necrosis factor α; VEGF: vascular endothelial growth factor*.

### Antivirals

#### Lopinavir/Ritonavir

Lopinavir/Ritonavir is a protease inhibitor approved for the treatment of HIV (108). Lopinavir was approved for the treatment of SARS-CoV during the epidemic of 2003 because it showed inhibitory activity against the virus *in vitro*. Lopinavir was also used against MERS-CoV because it has inhibitory activity against the virus both *in vitro* and in an animal models (109, 110). Lopinavir is used in combination with ritonavir because it increases the plasma half-life of lopinavir inhibiting the cytochrome P450 (111).

Despite these promising results, a Chinese clinical trial (ChiC-TR2000029308) in patients with SARS-CoV-2 infection showed that treatment with lopinavir–ritonavir added to standard supportive care was not associated with a statistically significant difference over standard care alone in the time to clinical improvement or mortality (87).

#### Hydroxychloroquine

SARS-CoV-2 needs an acidic endosomal pH for processing and internalization (8). *In vitro* data indicate that the antimalarial drug chloroquine exerts antiviral effects by increasing endosomal pH and abrogating virus-endosome fusion. Antiviral effects *in vivo* of hydroxychloroquine may be enhanced by the immune-modulating activity that this drug offers (112). Preliminary data suggests potential efficacy of hydroxychloroquine, particularly combined with azithromycin, in viral clearance. Hydroxychloroquine is often administered in conjunction with azithromycin, but caution is needed since these drugs are both associated with QT prolongation that could cause arrhythmias especially when combined with medications used to treat other chronic conditions (e.g., kidney failure, hepatic disease). In a small randomized study of 62 COVID-19 positive patients (not peer-reviewed) patients treated with hydroxychloroquine treatment showed an improvement in the clinical recovery and in the resolution of pneumonia compared to the control group (113). However, one observational study of 1,376 patients with COVID-19 treated with hydroxychloroquine showed no difference in the risk of being intubated or death compared to patients who did not receive hydroxychloroquine (88). The quick evolution of the COVID-19 pandemic and its associated mortality resulted in hasty publications occasionally not based on reliable data, which subsequently led to their retraction (114). Even when there is such sense of urgency, scrutiny and special attention to primary data would be prudent.

#### Favipiravir

Favipiravir is a drug approved for treatment of severe influenza virus infection in China. It is a new type of RNA-dependent RNA polymerase (RdRp) inhibitor. It inhibits viral polymerase activity because it can enter the cell and be recognized as a substrate by RNA polymerase when it is phosphoribosylated. It is capable of blocking the replication of several RNA virus (108). One randomized, controlled, open-label multicenter trial, showed no significant difference in disease recovery between 116 COVID-19 patients treated with favipiravir compared to 120 patients treated with arbidol, but the time of symptom improvement was shorter in favipiravir-treated individuals (not peer-reviewed) (89). Favipiravir is currently being tested in several clinic trials on COVID-19 patients.

#### Remdesivir

Remdesivir has broad-spectrum antiviral activity because it is an adenosine analog that can determine pre-mature termination of viral RNA (108, 112). It is currently being tested for treatment of Ebola virus infection and, in the future, might be useful to treat several other RNA virus infections (112, 115). Wang et al. (112) showed that viral infections in a human cell line, which is sensitive to SARS-CoV-2, could be inhibited by remdesivir.

In a cohort of 53 severely ill COVID-19 patients treated with remdesivir and observed for 18 days, 68% of patients improved in oxygen-support status, with a mortality of 13% overall (116). In a preliminary report of a randomized trial of 1,059 patients with COVID-19, those who received remdesivir had a faster recovery than patients who received a placebo (90). Goldman et al. (117) found that in 397 severe COVID-19 pneumonia patients without mechanical ventilation at baseline, there was no significant difference if they were treated for 5 or 10 days. However, in a randomized clinical trial of 158 patients, remdesivir was not associated with a significant clinical improvement compared to the placebo group comprised of 78 patients (91). Numerous clinical trials are ongoing to test remdesivir and its safety against COVID-19 infection.

#### Convalescent Plasma

The use of convalescent plasma was recommended as an empirical treatment during outbreaks of Ebola virus in 2014 and as a protocol for treatment of MERS (118).

Shen et al. (118) administered convalescent plasma transfusions to 5 patients with COVID-19 and ARDS. The donors had recovered from SARS-CoV-2 and had been asymptomatic for at least 10 days with documented anti-SARS-CoV-2 antibodies. In all patients, the neutralizing antibody titers significantly increased after plasma transfusion, the viral load declined, and the clinical conditions improved (118). One study of 10 patients infected by SARS-CoV-2 demonstrated the safety and efficacy of convalescent plasma transfusion, with improvement of clinical symptoms, reduction of pulmonary lesions, increase of lymphocytes count and titer of neutralizing antibodies, disappearance of SARS-CoV-2 RNA, and a better clinical outcome compared to 10 matched control patients (92).

Several other ongoing trials are testing the safety/efficacy profile of convalescent plasma transfusion in patients with SARS-CoV-2.

#### Camostat Mesylate

Camostat mesylate is a TMPRSS2 inhibitor. TMPRSS2 is a cellular protease that, together with ACE2, allows SARS-CoV-2 to enter target cells (13). Camostat mesylate, already approved for some forms of cancer and hepatitis, is being tested in ongoing clinical trial against COVID-19.

### Anti-inflammatory Agents

Early reports, mainly from China, suggested that over 60% of severely ill COVID-19 patients presented with additional organ dysfunction syndromes (119). This has been, at least in part, related to a sepsis-like syndrome induced by high levels of circulating cytokines. Cytokine storm may be induced by a superimposed septic syndrome or by the direct effect of the virus on the infected host (119). It has been suggested that anti-inflammatory drugs may ameliorate COVID-19 infections.

#### Steroids

The World Health Organization does not recommend the use of steroids because they could inhibit viral clearance and prolongate viremia (48). Stockman et al. (120) analyzed treatments used during the SARS outbreak of 2002–2003. They found that in 29 studies, use of steroids in 25 cases did not detect any efficacy and in 4 cases steroids could be harmful presenting side effects like delayed viral clearance, avascular necrosis, diabetes, and psychosis (33, 120). Therefore, systemic use of glucocorticoids need to be cautiously pursued (31). In a study of 548 patients with severe disease treated with high-dose corticosteroids, patients had an increased death rate than those not treated with corticosteroids (93). Some trials are exploring the effectiveness and safety of glucocorticoids in the treatment of COVID-19.

#### JAK-STAT Inhibitors

JAK-STAT inhibitors, like baricitinib, fedratinib, and ruxolitinib are potent anti-inflammatory drugs that are approved for rheumatoid arthritis and myelofibrosis. Patients infected with SARS-CoV-2 often present increased levels of pro-inflammatory cytokines and may benefit from the use of these drugs. A case series reported clinical improvement in COVID-19 patients treated with baricitinib (103). These drugs are currently being tested in multiple randomized controlled trials.

#### Intravenous Immunoglobulin

Intravenous immunoglobulin (IVIg) may reduce SARS-CoV-2-induced inflammatory response by blocking FcR activation on monocytes. There are several clinical trials that will evaluate the efficacy and safety of IVIg therapy in patients with pneumonia caused by SARS-CoV-2. In a retrospective study of 58 COVID-19 patients, the use of IVIg within 48 h of admission increased in-hospital recovery and reduced 28-day mortality rate (95).

#### Stem Cell Therapy

Mesenchymal stem cells (MSC) have immunomodulatory properties because they can inhibit T cell and macrophage activation and induce the formation of regulatory T cell and anti-inflammatory macrophages (121, 122). Their anti-inflammatory activity inhibits the secretion of IL-1, TNFα, IL-6, IL-12, and IFN-γ and promotes IL-10 and VEGF secretion which prevents lung tissue damage (121, 123). There is a pre-proof clinical trial that demonstrated the efficacy of the MSC treatment in patients with ARDS secondary to Influenza A (H7N9) infection (124). Several ongoing clinical trials are testing the mesenchymal stem cells therapy against SARS-CoV-2.

#### IL-6-Inhibitors

Tocilizumab, a drug used to treat rheumatoid arthritis, is a monoclonal antibody against the IL-6 receptor. Since elevated IL-6 levels are commonly found in COVID-19, tocilizumab is now under evaluation by a multicenter randomized controlled trial (ChiCTR2000029765). The preliminary clinical results are encouraging (48). In an uncontrolled study of 21 patients treated affected with severe COVID-19 infection, the use of tocilizumab improved symptoms and radiological findings (96). Numerous other studies are ongoing to test this drug in patients affected by COVID-19.

Other IL-6 inhibitors are being tested to treat COVID-19, including sarilumab and siltuximab. The latter showed a decrease in inflammatory markers in a study of 21 COVID-19 patients (not peer-reviewed) (104).

#### Ulinastatin

Ulinastatin is a serin protease inhibitor with anti-inflammatory effects approved in China and Japan for the treatment of acute pancreatitis and sepsis (123). There is an ongoing clinical trial (NCT04393311) that is testing the safety and efficacy of ulinastatin compared to placebo in COVID-19 patients.

#### Anakinra

Anakinra has been approved by the FDA for the treatment of rheumatoid arthritis and neonatal-onset multisystem inflammatory disease. It is a recombinant human interleukin-1 receptor antagonist (IL-1Ra), and it is currently being tested in ongoing trials with COVID-19 patients to contrast the uncontrolled inflammatory response. In one retrospective study of 29 patients with COVID-19, those that received high-dose of Anakinra in combination with standard treatment had a faster respiratory improvement compared to 16 patients who received standard therapy alone (72 and 50%, respectively) (97).

### RAS Inhibitors and Their Role in the Kidney?

The receptor for SARS-CoV-2, angiotensin-converting enzyme 2 (ACE2), is a membrane-bound aminopeptidase whose function is to cleave angiotensin I and angiotensin II into two peptides, angiotensin-(1–9), and angiotensin-(1–7) (8). Angiotensin-(1–9) has cardiovascular-protective effects and angiotensin-(1–7) acts as vasodilator and has anti-proliferative, and anti-fibrotic function (8, 125, 126).

Experimental animal models showed that, although ACE inhibitors and ARBs do not directly affect ACE2 activity, these agents can upregulate the expression and activity of the receptor in heart and kidney tissue (127). These drugs are commonly prescribed for patients with diabetes or cardiovascular disease who have higher risk of severe COVID-19 disease which has raised concern for the use of these drugs during the infection (8). However, the increased expression of ACE2 could limit the viral spread because it is not accompanied by an increase of TMPRSS2, so the virus could be tied to the receptor but could not enter the cells (8). Another possible protective role of ARBs in the lungs is mediated by angiotensin-(1–7) that has anti-proliferative and anti-fibrotic activity (8). Experimental animal models of acute lung injury, including a model of SARS-CoV infection, showed that ARBs reduce Ang II-mediated lung damage and therefore attenuate COVID-19 infection (127). In a retrospective study of 6,272 patients with COVID-19 and matched COVID-19 negative controls, infected patients were associated with increased use of RAAS inhibitors, but these drugs did not correlate with a more severe disease after multivariable adjustment (105). The study by Reynolds et al. (106) did not find any correlation between the use of anti-hypertensive drugs, such as RAS inhibitors (both angiotensin-receptor blockers and ACE inhibitors), calcium-channel blockers, beta-blockers or thiazide diuretics and the risk of infection or of having a severe disease in 5,894 patients.

The European and American Cardiology Societies, remarked that there is no evidence of a noxious effect of RAS inhibitors in patients affected by COVID-19 (128, 129). Moreover, discontinuation of RAS inhibitor both in healthy and infected individuals could be dangerous, especially in high-risk patients, so they suggest to maintain the ongoing therapy (130).

## Conclusions

SARS-CoV-2 effects on the kidney and in patient with underlying kidney disease is not well-characterized. Preliminary data has indicated that previous kidney disease could represent a risk factor, especially in elderly patients, for a more severe disease course. SARS-CoV-2 infects the kidneys and may induce acute kidney injury. While there is no current specific therapy, many drugs both antiviral and/or anti-inflammatory are being actively tested in randomized trials. Further studies are necessary to better understand disease pathology, acute kidney injury associated with infection, long-term renal consequences, and potential therapies. Rigorously controlled interventional studies and international registry analyses will be crucial to define risk factor and the best therapeutic approaches to resolving COVID-19 disease outcomes.

## Author Contributions

CB wrote manuscript the initial draft. MW, GZ, and LR participated in the manuscript writing. PC supervised the manuscript. All authors contributed to the article and approved the submitted version.

## Conflict of Interest

The authors declare that the research was conducted in the absence of any commercial or financial relationships that could be construed as a potential conflict of interest. The reviewer UM declared a past collaboration with one of the authors PC to the handling editor.
